# Co-regulated Transcripts Associated to Cooperating eSNPs Define Bi-fan Motifs in Human Gene Networks

**DOI:** 10.1371/journal.pgen.1004587

**Published:** 2014-09-11

**Authors:** Anat Kreimer, Itsik Pe'er

**Affiliations:** 1 Department of Biomedical Informatics, Columbia University, New York, New York, United States of America; 2 Center of Computational Biology and Bioinformatics, Columbia University, New York, New York, United States of America; 3 Department of Computer Science, Columbia University, New York, New York, United States of America; Yale School of Medicine, United States of America

## Abstract

Associations between the level of single transcripts and single corresponding genetic variants, expression single nucleotide polymorphisms (eSNPs), have been extensively studied and reported. However, most expression traits are complex, involving the cooperative action of multiple SNPs at different loci affecting multiple genes. Finding these cooperating eSNPs by exhaustive search has proven to be statistically challenging. In this paper we utilized availability of sequencing data with transcriptional profiles in the same cohorts to identify two kinds of usual suspects: eSNPs that alter coding sequences or eSNPs within the span of transcription factors (TFs). We utilize a computational framework for considering triplets, each comprised of a SNP and two associated genes. We examine pairs of triplets with such cooperating source eSNPs that are both associated with the same pair of target genes. We characterize such quartets through their genomic, topological and functional properties. We establish that this regulatory structure of cooperating quartets is frequent in real data, but is rarely observed in permutations. eSNP sources are mostly located on different chromosomes and away from their targets. In the majority of quartets, SNPs affect the expression of the two gene targets independently of one another, suggesting a mutually independent rather than a directionally dependent effect. Furthermore, the directions in which the minor allele count of the SNP affects gene expression within quartets are consistent, so that the two source eSNPs either both have the same effect on the target genes or both affect one gene in the opposite direction to the other. Same-effect eSNPs are observed more often than expected by chance. Cooperating quartets reported here in a human system might correspond to bi-fans, a known network motif of four nodes previously described in model organisms. Overall, our analysis offers insights regarding the fine motif structure of human regulatory networks.

## Author Summary

Previous analysis charts ubiquitous associations between the level of single transcripts and single corresponding genetic variants, eSNPs. However, most expression traits are complex, involving the cooperative action of multiple SNPs at different loci affecting multiple genes. The basic structure of variants within two *source* genes that affect the expression of two different *target* genes is compelling in terms of its potential to divulge information regarding the features of more complex interactions. We therefore devised a computational framework for the analysis of such variants, as ascertained from genomic sequencing data along with gene expression profiling of the same cohort. We focus on genetic markers that reside within interpretable regions of the genome: exon sequences, or transcription factors. Such cooperating eSNP sources are both associated with the same pair of target transcripts. We characterize such quartets through their genomic, topological and functional properties. Our findings suggest that this regulatory structure of quartets exhibits distinct characteristics and is frequent in real data, but is rarely observed in permutations.

## Introduction

Markers associated with changes in gene expression, called eSNPs have been extensively mapped using high throughput genomic data [2], [3], [4], [5], [6], [7], [8], [9], [10]. They allow effectively delineating regulatory associations between each eSNP source and each of its regulated target transcripts. Taken together, these source-target links comprise a regulatory network that abstracts both the genes at source loci as well as their targets as nodes.

Regulatory networks have been characterized as featuring specific motifs as their fundamental building blocks [11], [12]. These motifs occur significantly more than expected by chance and suggest respective functional mechanisms. Specifically, studies in model organisms highlighted the bi-fan motif which consists of two regulators regulating two genes as having a functional role, e.g. of a filter and synchronizer of feedback loop signals [12], [13]. While previously studied networks are often derived from TF-DNA or protein-protein binding experiments, this work utilizes genetics-genomics data to study the bi-fan motif across a human regulatory network.

Model organisms, amenable to pervasive experimental methods, suggest regulatory networks to commonly include structures more complex than single SNP – single gene links, e.g. mapping genetic interactions in yeast [14], [15]. In humans, where experimental approaches are more limited, eSNPs provide natural perturbations that inform us of similar regulatory links and systems. Concerted analysis of a multitude of eSNPs allows better understanding of the interactions that establish their network structure. Statistically, epistatic interaction is defined as the deviation from additivity in a linear model involving two or more loci [16], [17]. Unfortunately, finding such association signal for statistical interaction between a pair of SNPs in even a single phenotype has proven computationally difficult [15], [18], [19], [20]. Association analysis across all pairs of SNPs vs. all transcripts exacerbates this tractability problem.

While structures of multiple eSNPs to one transcript offer one lens for genetic-genomic analysis, a complementary perspective is provided by regulatory modules, where a single eSNP is associated to multiple genes [1], [21], [22]. Modularity of gene regulatory networks was shown to be a major organizing principle of biological systems [23], with modules often defining functional units of a biological network: each such units consists of a set of elements (e.g. genes) working jointly to perform a distinct function.

Analysis of single eSNP-single transcript interactions indicates that variation in genomic DNA can affect transcription in multiple ways. Level of transcripts *in cis* of an eSNP may be altered due to allelic variation in *cis*-regulatory elements [24], while *trans* association can, for example, be the result of an eSNP in a transcription factor that regulates the expression of its distal targets transcripts. Associations in *cis* are easier to detect because of favorable testing burden. Unfortunately, such associations are limited in their capacity to inform us regarding the network of regulatory interactions between one gene and another, as both the eSNP and the transcript are from the same locus. In contrast, *trans* eSNPs can identify downstream effects and previously un-annotated regulatory pathways. Moreover, when considering independent association between more than a single eSNP and more than a single gene, the genomic distances between eSNP sources and their gene targets require special attention. In the case of examining a pair of proximal eSNPs, their frequent co-inheritance would induce statistical dependence (linkage disequilibrium) between them. Thus, for most independent pairs of eSNPs that cooperate in regulating the same transcript, at least one of them will have a *trans* effect.

In our previous work [1], we studied eSNPs associated with simplest modular unit of two transcripts, together creating a *triplet*. We focus on mutually independent triplets, whereby the eSNP association with either of the two transcript remains nominally significant given the respective other transcript, as well as and directionally independent triplets, where only one of these association signals remains nominally significant given the level of the other transcript. We established the occurrence of such triplets in real data significantly more than expected by chance.

In this study, we devise a computational framework for examining pairs of triplets that share the same associated two genes. We hypothesize that such eSNP-transcript *quartets* will highlight true eSNP associations, and demonstrate that by analyzing their distinct topological and functional properties. These properties differ significantly from those of spurious quartets with candidate association signals. Moreover, we replicated those properties in an independent dataset with a larger number of samples [4], supporting the robustness of our findings. In particular, the two eSNPs in a quartet tend to have independent, but consistent effect on the pair of genes they co-regulate.

## Results

### Computational framework for associating pairs of SNPs with pairs of genes

#### Definition and discovery of quartets

We used a publicly available classic dataset of 50 fully sequenced Yoruban samples [25] along with their transcription profiles from RNA-seq data [26], bearing in mind that such available cohorts are limited in size. Due to this small sample size, we have limited power in detecting association. Therefore, most candidate eSNPs can only be designated as such with various levels of uncertainty. We demonstrate the ability to recapitulate the observed phenomena in a larger dataset [4] using the same method.

**Figure 1:**
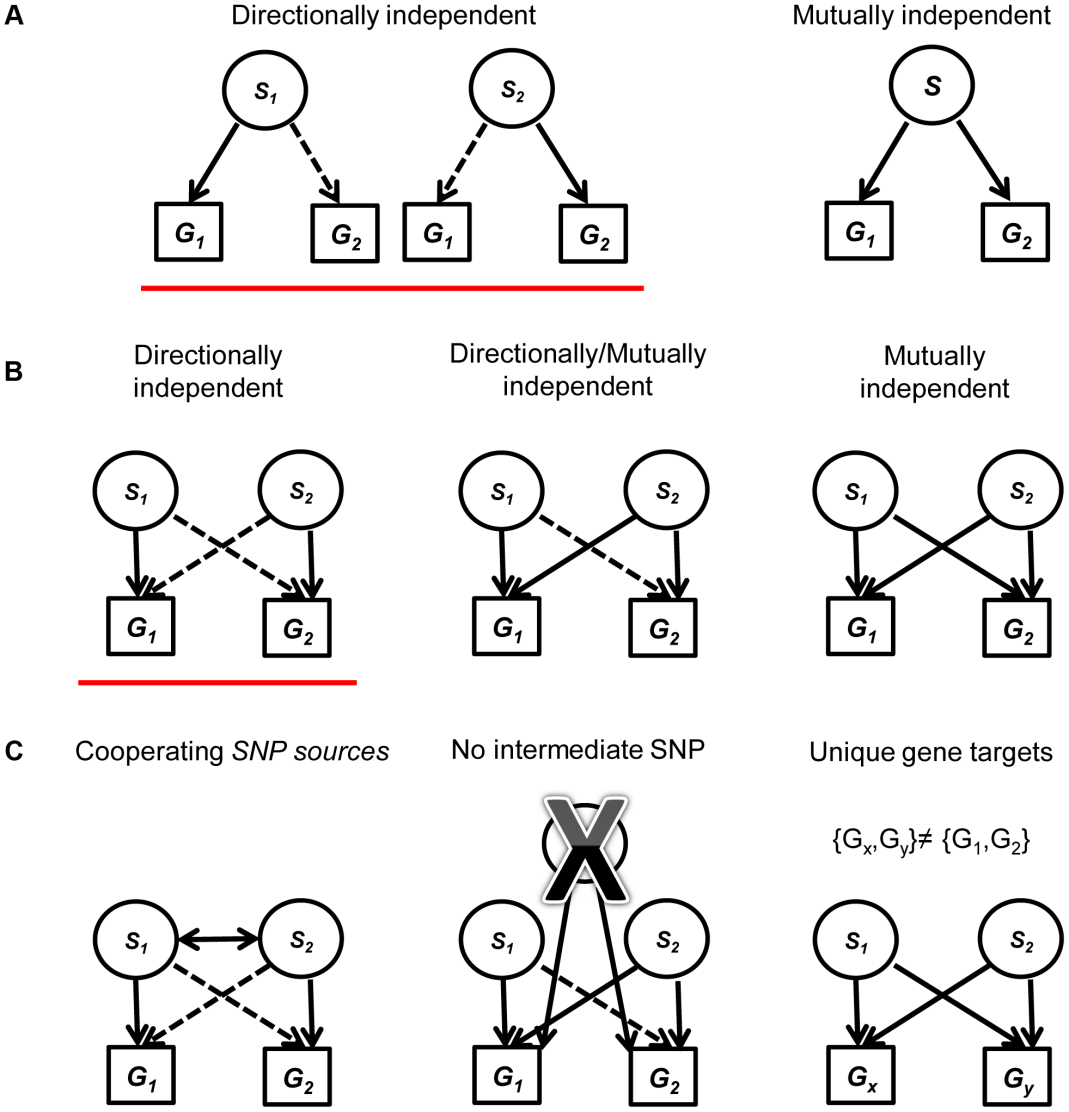
A diagram explaining the framework for creating and filtering quartets. (a) We include mutually independent and directionally dependent triplets. A solid line represents mutually independent association. A dashed line represents directionally independent association. (b) Quartets are assembled from triplets in (a) with the same associated gene targets. Quartets are assembled either from two directionally independent triplets (red underline), two mutually independent triplets or one directionally independent triplet and one mutually independent triplet. (c) We filter the quartets using three criteria: (1) Restricting our analysis to quartets with cooperating eSNPs sources, i.e., SNPs that carry independent information towards predicting the expression of each one of the two genes. (2) Removing quartets where a third intermediate SNP can explain the expression to either transcript better. (3) Focus on quartets that have two unique gene targets, i.e., after filtering, no quartet has the same pair of gene targets.

We evaluated two categories of candidate eSNPs that reside within regions along the genome with known regulatory potential, i.e., within the span of known exons and TFs (including introns) (Figure S1 in Text S1; see Methods). These eSNPs can be associated with the expression of both local and distal genes. We consider all mutually independent and directionally independent triplets (Figure 1a, see [1] for details). Going beyond the associations of a single eSNP *source* requires the examination of pairs of triplets that share the same *target* transcripts. We call this arrangement a *quartet* (Figure 1b). We aim to study quartets with *cooperating* eSNP *sources*, i.e. SNPs that carry independent information towards predicting the level of each one of the two transcripts, and no third intermediate SNP can explain the expression to either gene better (Figure 1c; see Methods). We note that such *cooperating quartets* may overlap in their genes, introducing double-counting of the same effect in different quartets. To ensure our analysis involves quartets with distinct targets, we filtered this set of cooperating quartets further and focused on the quartets that have two unique gene targets. In this workflow, no post-filter quartet has the same pair of gene targets as any other (Figure 1c).

#### Evidence for the validity of quartets

We choose an association testing threshold of 10^−4^ (Figure S2 in Text S1) by the number of quartets produced, aiming at FDR<5% when comparing to the number of quartets in permutations. We examined the number of triplets in real data vs. 100 permuted data sets where sample labels had been switched. In permuted data sets, an average number of 33,329 triplets exceeded association p-value threshold of 10^−4^ (Figure S3 in Text S1; see Methods). We therefore considered a comparable set of triplets, the same number of top results in real data, which corresponded to an association p-value threshold of 10^−4.52^. This step creates an equal starting point for permuted vs. real datasets when approaching further analysis. We next examined the number of quartets formed by such triplets in real vs. permuted datasets. We observe that the number of 47,006 quartets in real data is consistent with chance expectations (empirical p-value = 0.07, Figure S4 in Text S1). Out of 47,006 quartets, there are 4,009 quartets with unique gene targets.

**Figure 2:**
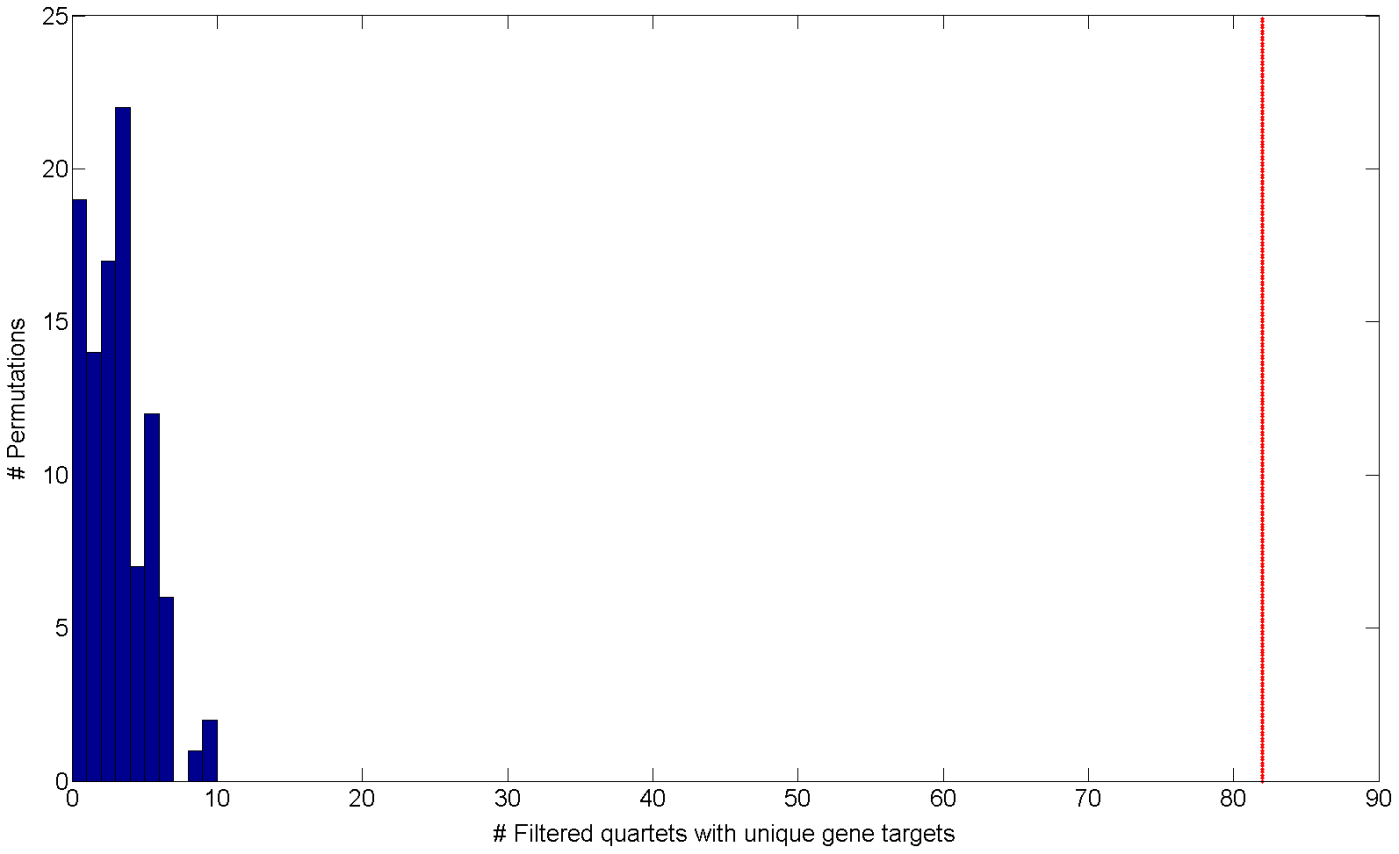
Histogram of the number of filtered quartets with unique gene targets in 100 permutations. The red line indicates the observed number of filtered quartets with unique gene targets in real data (empirical FDR<5%).

Interestingly, when examining cooperating quartets, we observe 374 such quartets in real data (0.8%) comparing to a mean of 19.18 in permutations (0.063% out of a mean of 30,250 quartets) (Figure S5 in Text S1). These results establish that the regulatory structure of cooperating quartets is nearly exclusive to real data, as it is rarely emerges in permutations. Out of 374 cooperating quartets with cooperating eSNP sources we focus on the 82 quartets that have two unique gene targets (Table S1). These include 2.05% of the total of 4,009 quartets with unique gene targets. Such unique cooperating quartets are more common in real data than in permuted data both in absolute number as well as in their relative fraction: permutations include only 3.71 such quartets on average (empirical FDR<5% , Figure 2) 0.097% of an average of 3,819 quartets with unique gene targets).

Cooperating quartets are a motif of the human regulatory network analogous to the bi-fan motif found in *E. coli* [12], [13]. We set out to characterize these cooperating quartets and study their functional, genomic and topological properties. In the next section we compare such quartet properties to permuted data, highlighting the quartets observed in real data as a true phenomenon, as opposed to quartets observed by chance.

#### Quartets in real data have distinct properties

Since the number of quartets in each permutation is low (Figure 2), we combine all quartets across all permutations and treat them as a “permuted set” of 342 quartets. From this point we compare the 82 quartets in real data vs. those in the permuted set to uncover properties that are unique to real structures.

### Distribution of genomic properties of eSNP sources and their gene targets

**Figure 3:**
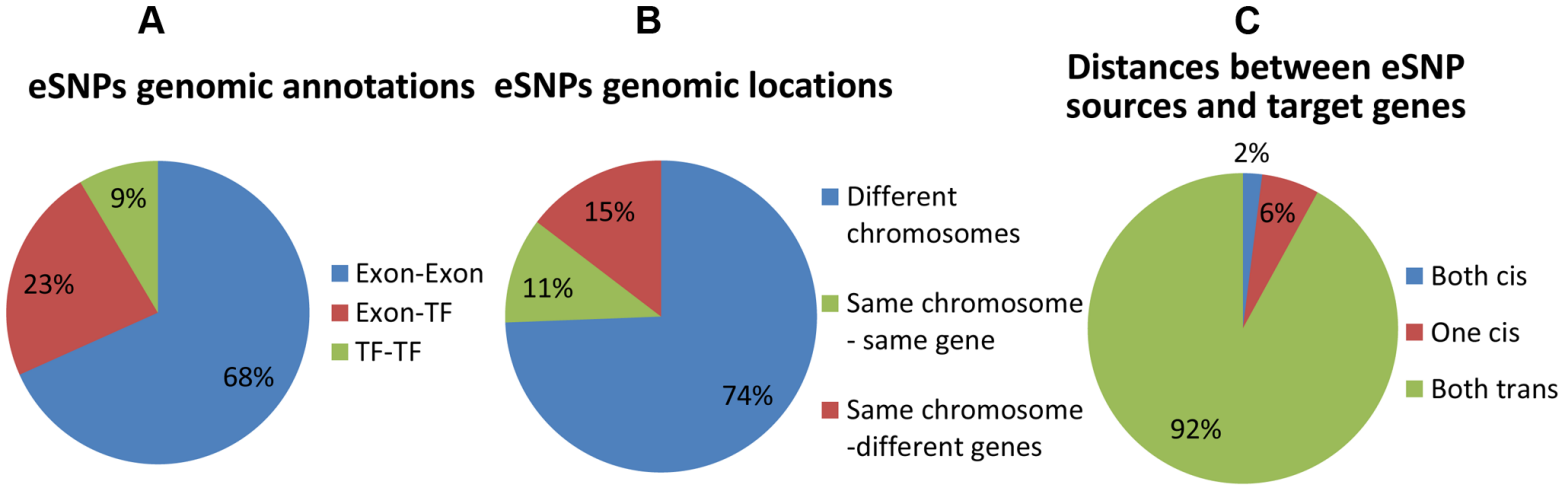
Distribution of genomic properties of eSNP sources. By (a) genomic annotation (b) relative genomic location (c) distances between them and their targets. An eSNP is said to be *in cis* if it resides within the span of the target gene and *in trans* otherwise.

We first record genomic annotation categories of eSNP sources (Figure 3 and Figure S6 in Text S1). eSNP sources tend to be one in exon and one in TF (Figure 3a; Fisher's exact *p*<1.9×10^−8^ compared to the permuted set, see Figure S6a in Text S1), or both in exons (Fisher's exact *p*<0.013 compared to permuted set). We notice that most eSNP sources are located on different chromosomes (74% Figure 3b). For comparison, there are only 3.8% of eSNP sources on different chromosomes in the permuted set (13 out of 342; Figure S6b in Text S1). An eSNP is said to be in *cis* of a target if it resides within the span of the target, and in *trans* otherwise. We characterize the *cis/trans* regulation of the four pairs of eSNP sources and their gene targets in each quartet by binning quartet data into three *cis*/*trans* categories: (1) two *cis* relationships (2) one *cis* relationship (3) two *trans* relationships. We notice that only a fraction of quartets involves *cis* regulation (Figure 3c), compared to none in the permuted set (Figure S6c in Text S1). The target genes are located mostly (83%) on different chromosomes which is consistent with empirical expectation based on permutation. They are observed to be co-expressed significantly (*p*<4×10^−11^) more often than in real data when comparing the absolute value of the correlation coefficient.

These results highlight unique properties of cooperating eSNPs and their distances from target transcripts. Specifically, we show that pairs of eSNP sources are located on different chromosomes.

### Characterizing dependencies within cooperating quartets

**Figure 4:**
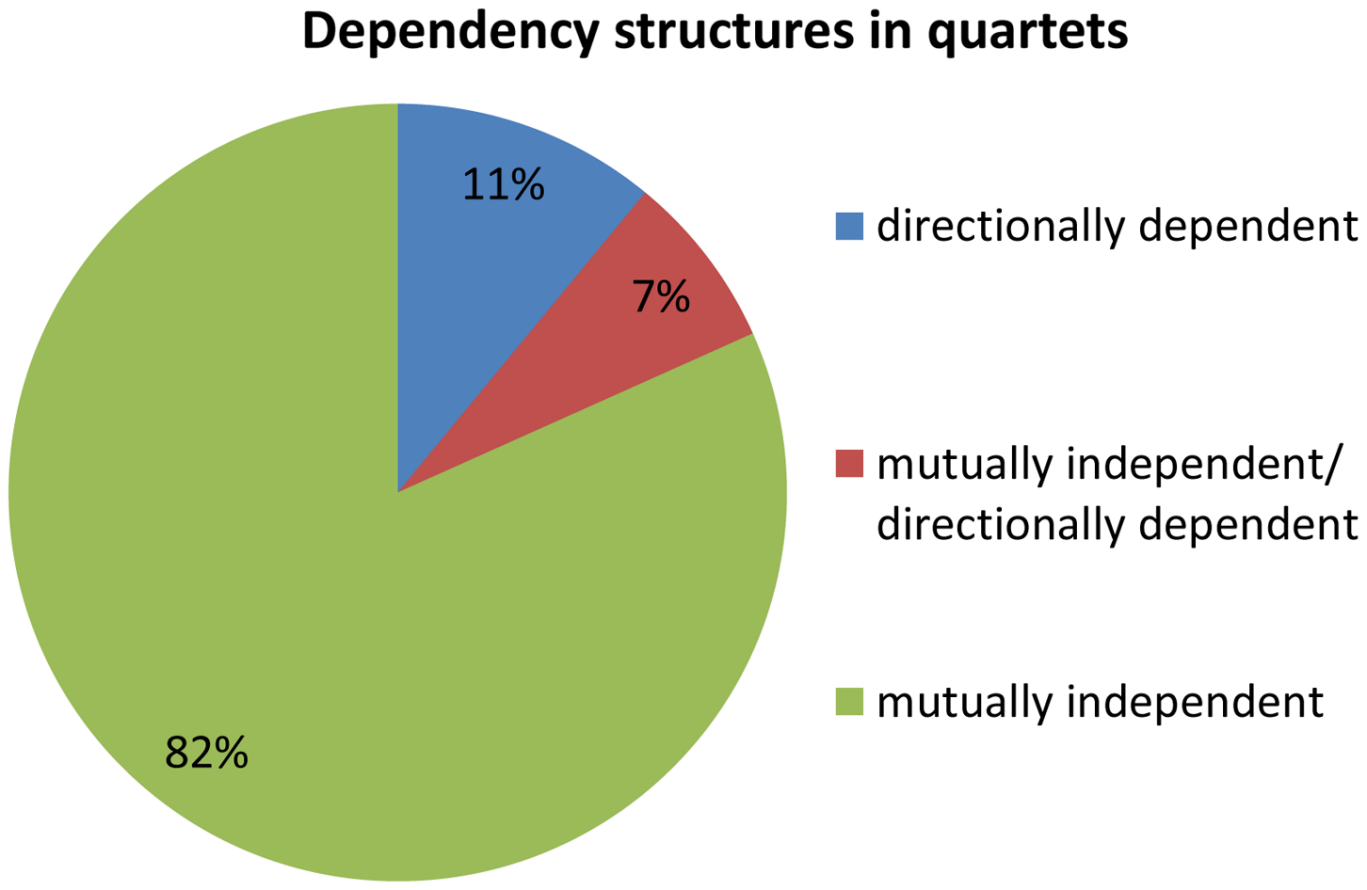
Dependency structures in quartets. Quartets are either comprised of a pair of mutually independent association signals, one directionally dependent association and one mutually independent association, or a pair of directionally dependent association signals.

We examine the dependency across association signals for each quartet source, i.e., whether the effect is mutually independent or directionally dependent. Dependencies within a quartet are therefore either (1) pair of mutually independent associations (2) one directionally dependent association and one mutually independent association, or (3) a pair of directionally dependent associations. We observe that 82% (67 out of 82) of the quartets are composed of a pair of mutually independent associations (Figure 4). This is significantly more than expected according to the permuted set, that includes mostly quartets with a pair of directionally dependent associations (Fisher's exact *p*<2.3×10^−35^, Figure S7 in Text S1). These results suggest that the eSNP sources affect the expression levels of both transcripts in a mutually independent manner rather than through directional dependence.

### Identifying direction of effect between eSNP sources and gene targets

**Figure 5:**
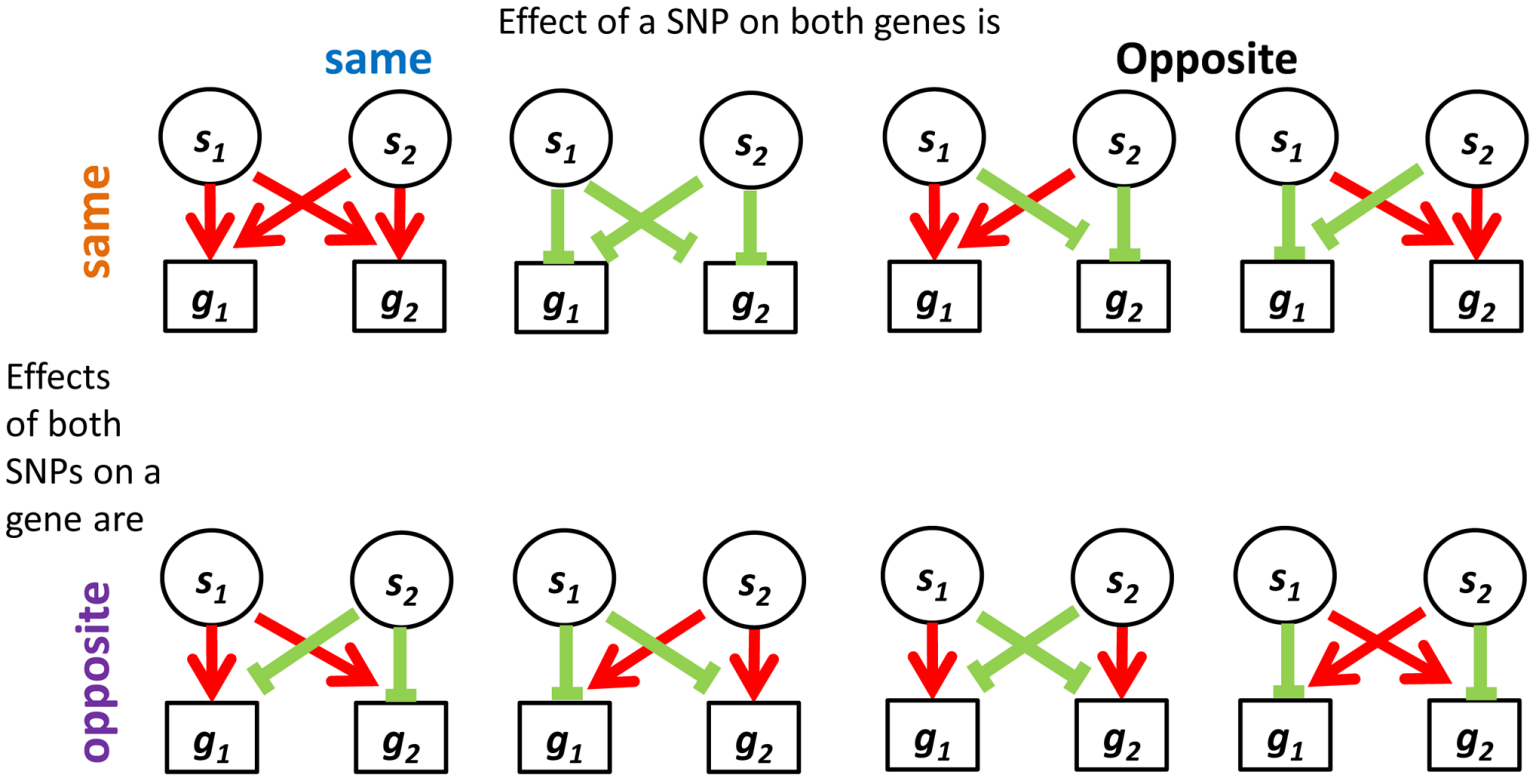
Four categories describing the eight configurations of directions of effect observed in real data between eSNP sources and gene targets. Both SNPs can have the same or opposite effect on a gene and both genes may experience the same or opposite effect of a SNP.

We were interested in examining the direction of SNP effects on gene expression. Within quartets we orient all SNP effects by using the convention of up (down) regulation to mean positive (negative) correlation between the number of copies of the minor SNP allele and the expression level of the associated gene. Out of the 2^4^ = 16 up/down configurations that are theoretically possible between two sources and two targets, we observe only eight configurations in real data – the ones with an even number of “up” effects (Figure 5). Consideration of the symmetry between the two sources, as well as the one between the two targets, highlights a sense in which these eight categories involve *consistent* directions of effect, as we now explain. It is natural to classify the categories into four pairs, each defined by two binary criteria. The first criterion considers whether the two source SNPs have the same directions of effect on one gene as they do on the other or whether directions of effect on the second gene are opposite to the first one. The second criterion distinguishes whether the effect of one SNP on the two target genes is in the same direction as the effect the other SNP has on them, or whether directions of effect of the second SNP are opposite (Figure S8a in Text S1).

In contrast to the real data, where all quartets are consistent, 30% (101 of 342) of quartets in the permuted set are *inconsistent* quartets (Figure S8b in Text S1), meaning that the effects of the two SNPs on one of the targets go in the same direction, while their effects on the other target are opposite (Figure S9 in Text S1).

We hypothesized that quartets in real data may be practically forced to be consistent due to correlation patterns across the expression levels of their targets. Specifically, a source SNP would the same (opposite) effect on both target genes due to their expression being correlated (anti-correlated). Indeed, we observe this pattern across all quartets in the real data but not always in the permuted set.

**Figure 6:**
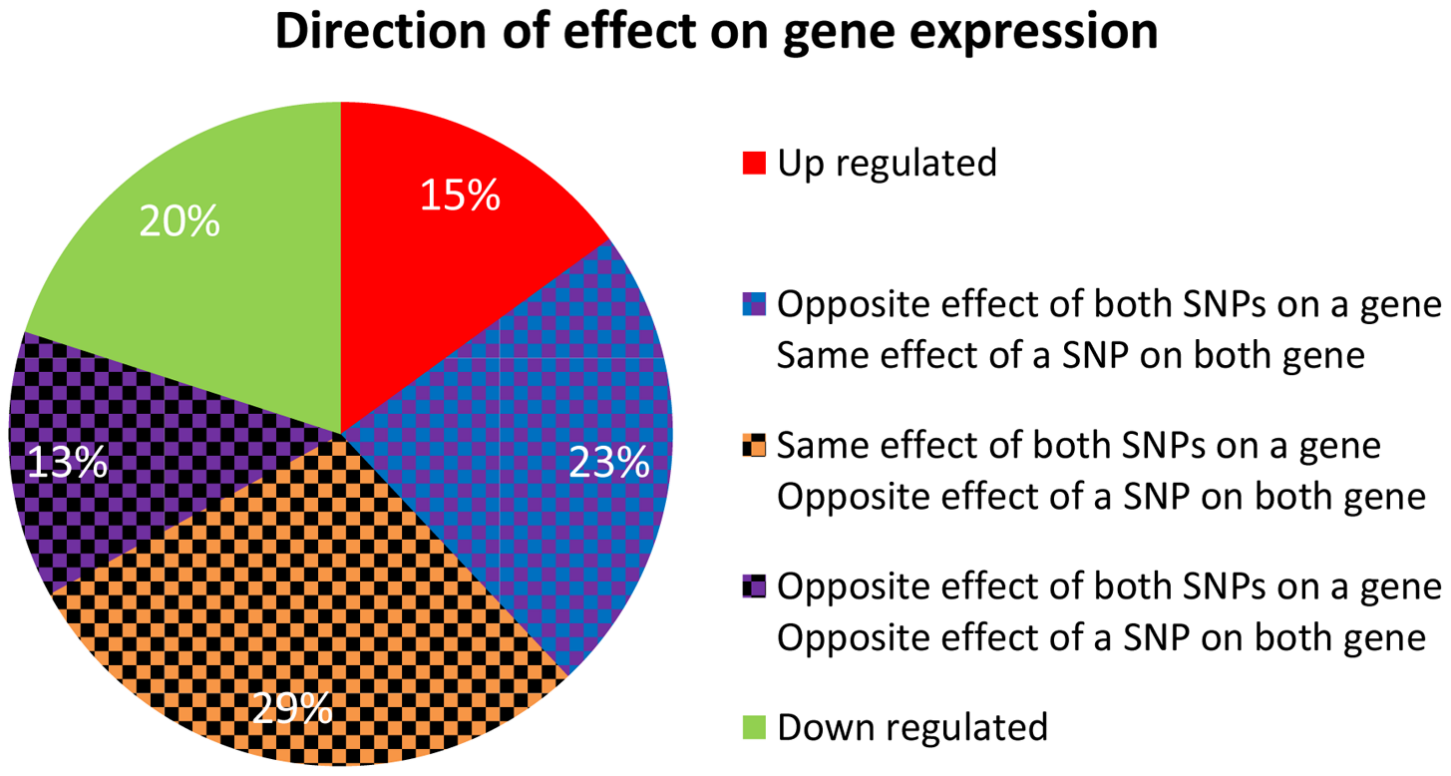
Direction of effect for eSNP sources association with gene targets expression. In real data when the eSNP sources are located on different chromosomes. Both SNPs can have either the same or opposite effect on gene targets. The effect of a SNP on both genes is either the same or opposite.

There are a couple of statistical challenges involved in comparison of real quartets to those observed in permutations (Note S1 in Text S1). When these are addresses, specifically by analyzing eSNPs sources from the same quartet but from different chromosomes, we observe them to be enriched for same-direction effects compared to their permuted set counterparts (Figure 6 and Figure S8c in Text S1) and the gene targets to be located on different chromosomes. We listed all characterizing features of cooperating quartets (Table S1).

**Figure 7:**
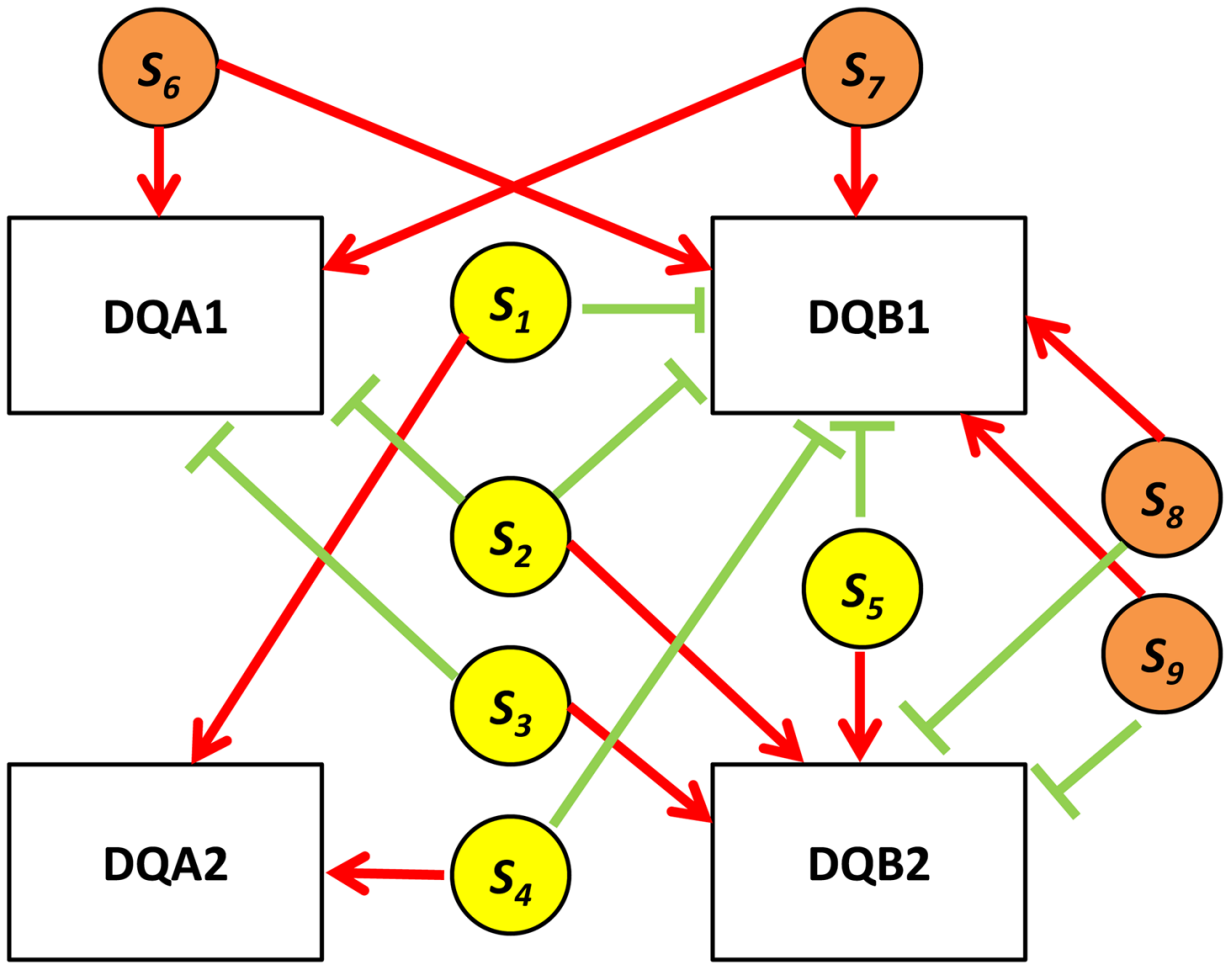
HLA quartet. An example of examining eSNP sources and gene targets on the same chromosome together. Assembled quartets at the HLA locus highlight a 9-SNP haplotype associated with co-expression of DQA1-DQB1 and anti-correlated to DQA2-DQB2. s1, s2, s3, s4 and s5 (yellow circles) correspond to rs2808143, rs1129740, rs9274634, rs9274389 and rs1142334 respectively. s6, s7, s8 and s9 (orange circles) correspond to rs8227, rs1130034, rs9272851 and rs1130116 respectively. Red edges indicate up-regulation, green edges indicate down-regulation.

A particularly illustrative sub-group of 7 quartets includes those with eSNP sources and gene targets along the MHC region of chromosome 6 (Table S1). This is significantly more (Fisher's exact p<0.0014) than 4 out 342 (∼1%) in the permuted set. The eSNP sources collapse to reference alleles of rs9274634, rs1129740, rs1142334, rs9274389 and rs2808143 and non-reference alleles of rs1130034, rs8227, rs1130116 and rs9272851 downregulating HLA-DQA1 and HLA-DQB1 and upregulating HLA-DQA2 and HLA-DQB2. These common variants are shared by specific assembled sequences (Table S3 in Text S1). All the genes containing eSNP sources and target genes are collapsed into the following four HLA genes: HLA-DQB1, HLA-DQA1, HLA-DQB2 and HLA-DQA2 (Figure **7**). All four genes are involved with the MHC class II receptor activity (enrichment FDR<1.4.10^−12^), and serve as an example how quartet structures create functional units.

### Functional enrichment of quartets

We perform a gene set enrichment analysis to examine if the pair of gene targets shares a GO category significantly more than pairs in the permuted set. In this case we observe a higher number of shared descriptors which is not significant in this dataset (Fisher exact p-value<0.14). Interestingly, when we focus the enrichment analysis on pairs of genes that harbor cooperating SNP sources, we observe a significant difference (Fisher exact p-value<1.5×10^−6^). This supports our ability to detect SNPs that cooperate together to perform a joint function. We were intrigued to examine if our approach could be applied to understand gene regulatory networks underlying complex diseases. We therefore utilized the GWAS catalog [27] to find all genes that harbor a GWAS SNPs in our dataset. We then intersected this list with the genes that harbor cooperating SNPs in real data and compared to permutations. We observe a significant overlap of GWAS loci with at least one eSNP source, for quartets with sources that reside on different genes (Fisher exact p-value<0.017). This indicates that our approach could shed light on regulatory circuits that are involved in complex disease. For example, in quartet #35 (Table S2 in Text S1) eSNP sources rs16877111 and rs7925000 are on chr5 and chr11 respectively. The eSNP sources reside in genes CMYA5 and RPL27A which are obesity GWAS loci. The gene targets HIST1H1D and HIST1H2AH are part of a histone cluster on chr 6.

### Replication of quartet properties in a larger dataset

Since our initial study was underpowered, we attempted to replicate the discovered properties of cooperating quartets in a larger, more recent dataset. We hypothesized that the fraction of true positives among signals of association to be higher is such a dataset, thereby pointing to true characteristics of quartets, rather than potential artifacts of false positive signals. We repeat our analysis in the Geuvadis [4] dataset for each of its five populations: Utah European (CEU; n = 91), Finnish (FIN, n = 95), British (GBR; n = 94), Italian (TSI; n = 93) and Yoruban (YRI; n = 89) as well as on the combined set of all European samples (n = 373). We observe that the number of association signals achieving p-value<10^−5^ is enriched in true positive associations (∼5 fold more associations than expected). Overall, we replicate all properties (Same effect of both eSNPs, distal regulation, eSNP sources on different chromosomes, gene targets on different chromosomes and consistency of quartets) that were found in the smaller dataset, most of them at higher frequencies (Table S2 in Text S1). This provides an additional support from an independent dataset to the validity of quartets and their characteristics.

## Discussion

Discovering the building blocks of regulatory network has been an active field of research in the last decade [12], [28]. Specifically, the human regulatory network was the focus of a multiple recent studies involving diverse data types [11], [29]. In this work we devised a computational framework to study characteristics of cooperating quartets comprised of a pair of cooperating eSNP sources that reside either in exons or in the span of TFs, and a pair of associated target transcripts.

Our results establish that the regulatory structure of cooperating quartets is nearly exclusive to real data, and exhibits unique functional, genomic and topological characteristics. Cooperating quartets reported here in a human system might correspond to bi-fans, a known network motif of four nodes, previously described in model organisms [12].

Most cooperating quartets involve pairs of eSNP sources located on different chromosomes, away from their targets, which are themselves mostly located on different chromosomes. These quartets typically comprise of a pair of mutually independent association signals. All quartets are consistent in terms of the direction of eSNP effects on correlated and anti-correlated transcripts. We identify a separate sub-group of quartets with eSNP sources and gene targets all involving 4 MCH Class II genes from chromosome 6, highlighting a functional unit built from the quartet motif.

This study holds the promise for extension beyond its current limitations. First, our focus on causal variants localized to the single-base resolution imposed relying on a dataset of fully sequenced individuals along with their transcription profiles. Such cohort sizes are limited in size, reducing the power to detect association and allowing us to observe only the strongest effects. Potential increase in sample size for expression quantitative trait loci (eQTL) data would enable detection of eSNP associations and regulatory motifs at greater significance and confidence. Second, the current analysis focuses on discovering a network motif where pairs of transcripts are co-regulated by a pair of variants. Mining the data for additional motifs can elucidate other structures in the human regulatory network. Overall, both the raw datasets [25], [26] and supporting databases [30], [31], [32], [33] in this work were noisy and limited. As functional annotation continues to build up, better understanding of motifs would be facilitated.

In this and in our previous work [1] we define network motifs showing them to be prevalent in real data, explaining the organization of *trans* regulation. Comparison of such structures between healthy and affected samples and across different tissues is likely to improve understanding of disease and developmental regulatory processes. Future studies could expand this approach to focus on complex disease circuits by using this framework on a dataset that is focused on GWAS SNPs and find quartets where the eSNP sources are also known GWAS loci.

The vast majority of eQTL studies involve analyses that are based on considering a single SNP associated with a single transcript, primarily *in cis* [4], [26], [34], [35]. While these analyses capture only a fraction of genetic contribution to changes in the regulatory landscape, the advantage is high statistical power for detecting associations. A complementary effort focuses on building networks from eSNP data [21], [36], [37], [38]. While these studies provide much more comprehensive models, they lack the same strength of statistical assurance in their findings. The main advantage of our approach is that it provides a unique framework for analyzing eSNP data by bridging these two approaches, establishing statistical guarantees on our inferred results using permutations. Applying such analysis to different datasets can shed light on the architecture of the human regulatory network and the role genetics plays in shaping it.

## Materials and Methods

### Data details and processing

We analyze a cohort of 50 Yoruban samples, for which genotypes of SNVs that are fully ascertained from sequencing data [25] along with RNA-seq data [26] are publicly available. Briefly, the raw dataset consists of 10,553,953 genotyped SNVs and expression measurements (quantile-quantile normalized values) of 18,147 genes with Ensembl gene ID across these 50 samples. Standard filters have been applied to the genetic data: Minor allele frequency >0.05, SNP missingness rate <0.1 and individual missingness rate <0.1 [39]. After filtering, data for analysis consists of 50 samples with 7,206,056 SNPs. The Geuvadis [4] dataset that we use for replication consists of five populations: Utah European (CEU; n = 91), Finnish (FIN, n = 95), British (GBR; n = 94), Italian (TSI; n = 93) and Yoruban (YRI; n = 89) as well as on the combined set of all European samples (n = 373). After filtering all SNPs with Minor allele frequency <0.05 and focusing only on SNPs in exons and TFs, there are 42810, 43561, 43279, 43214, 61960 and 43365 for CEU, FIN, GBR, TSI, YRI and EUR respectively.

### Association testing

For association analysis, we consider only SNPs that reside within candidate regulatory regions along the genome. In Kreimer et al. [1] we detect enrichment in *trans* association signals for eSNPs in exons and in TFs in this dataset. For TFs, the number of multiple associated transcripts is significantly higher for TFs in the real dataset than in permuted data sets. For exons, there is an excess of the number of eSNPs within exons indicating true positive results. We test for association between a SNP and every gene; we consider SNPs within the span of known exons and TFs (including introns) [40], [41]. We test for association using linear regression performed by the –assoc command in PLINK [39].

### Obtaining a random distribution of association test-statistics

Examining the random distribution of association tests is helpful in evaluating the empirical significance of results. This is achieved by generating 100 permutations that shuffle the sample IDs. This allows repeating the analysis of genotypes vs. expression on permuted data while maintaining the correlation structure among the genotype profiles and among the expression profiles, separately.

### Creating and filtering quartets

We assemble quartets from directionally and mutually independent triplets that consist of a SNP and two associated genes. A mutually independent triplet is when both of the association pairs remain nominally significant given the respective other gene and a directionally independent triplet is where only one of the association pairs remain nominally significant given the other gene. Two triplets that share the same associated genes define a quartet. We then filter these quartets further using the following rules:

1. We are only interested in quartets where both SNPs carry significant information in predicting the expression of gene 1 and gene 2. i.e. 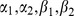 should be significantly different than zero.
  g_1_ – represents the expression of gene 1.
  g_2_ – represents the expression of gene 2.
  s_1_ – represents the minor allele count SNP 1.
  s_2_ – represents the minor allele count SNP 2.
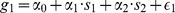


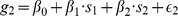

2. Moreover, we are interested in examining quartets that have no intermediate third SNP (s_3_) that can explain the expression better.
  The third intermediate SNP should satisfy the following:
  1. On the same chr

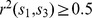
 and 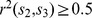

  3. s_3_ should be in a triplet with the two genes.

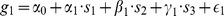


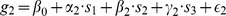


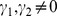

### Software

The code for all methods presented in this thesis can be found in the following link: http://www.columbia.edu/~ak2996/Software.htm

## Supporting Information

Table S1 A comprehensive description of 82 cooperating quartets. 3000000000 corresponds to different chromosomes.
(XLSX)

Text S1 Supplementary file containing all supplementary figures and text and some of the supplementary tables. Figure S1: Illustrating the association testing between pairs of SNPs within known regulatory regions and genes (close or distal): regulatory elements appear in red. If the regulatory element has a SNP within the boundaries of an exon or a TF then we check for association (p<10^−4^ denoted by a red edge) using linear regression between the minor allele count of the SNP and any gene. Figure S2: Histogram of the number of association pairs in 100 permutations for a p-value cutoff 10^−4^. The red line indicates this number in the real data. Figure S3: Histogram of the number of triplets in 100 permutations, at association p-value of 10^−4^. The red line indicates the observed number of triplets in real data at association p-value 10^−4.52^. Figure S4: Histogram of the number of quartets in 100 permutations, at association p-value of 10^−4^. The red line indicates the observed number of quartets in real data at association p-value 10^−4.52^. Figure S5: Histogram of the number of filtered quartets in 100 permutations, at association p-value of 10^−4^. The red line indicates the observed number of filtered quartets in real data at association p-value 10^−4.52^. Figure S6: Distribution of genomic properties of eSNP sources in the permuted set: by (a) genomic annotation (b) relative genomic location (c) distances between them and their targets. An eSNP is said to be in cis if it resides within the span of the target gene and in trans otherwise. Figure S7: Dependency structures in quartets of the permuted set: Quartets are either comprised of a pair of mutually independent association signals, one directionally dependent association and one mutually independent association, or a pair of directionally dependent association signals. Figure S8: (a) Direction of effect for eSNP sources association with gene targets expression in (a) real data (b) permutations (c) permutations when the eSNP sources are located on different chromosomes. Both SNPs can have either the same or opposite effect on gene targets. The effect of a SNP on both genes is either the same or opposite. Figure S9: All eight regulation patterns of inconsistent quartets. Note S1: Statistical challenges in comparing real vs. permuted quartets. Table S2: Replication of quartets' properties in the Geuvadis dataset [38]. For each property in the first row we indicate the percentage in the original, smaller dataset. Table S3: The distribution of HLA common variants in specifically assembled sequences.
(DOCX)
