# Supplementary material for: Co-regulated Transcripts Associated to Cooperating eSNPs Define Bi-fan Motifs in Human Gene Networks
Source: PLoS Genet. 2014 Sep 11;10(9):e1004587. doi: 10.1371/journal.pgen.1004587 (PMC4161301; doi:10.1371/journal.pgen.1004587)
Supplement: Text S1 — Supplementary file containing all supplementary figures and text and some of the supplementary tables. Figure S1: Illustrating the association testing between pairs of SNPs within known regulatory regions and genes (close or distal): regulatory elements appear in red. If the regulatory element has a SNP within the boundaries of an exon or a TF then we check for association (p<10−4 denoted by a red edge) using linear regression between the minor allele count of the SNP and any gene. Figure S2: Histogram of the number of association pairs in 100 permutations for a p-value cutoff 10−4. The red line indicates this number in the real data. Figure S3: Histogram of the number of triplets in 100 permutations, at association p-value of 10−4. The red line indicates the observed number of triplets in real data at association p-value 10−4.52. Figure S4: Histogram of the number of quartets in 100 permutations, at association p-value of 10−4. The red line indicates the observed number of quartets in real data at association p-value 10−4.52. Figure S5: Histogram of the number of filtered quartets in 100 permutations, at association p-value of 10−4. The red line indicates the observed number of filtered quartets in real data at association p-value 10−4.52. Figure S6: Distribution of genomic properties of eSNP sources in the permuted set: by (a) genomic annotation (b) relative genomic location (c) distances between them and their targets. An eSNP is said to be in cis if it resides within the span of the target gene and in trans otherwise. Figure S7: Dependency structures in quartets of the permuted set: Quartets are either comprised of a pair of mutually independent association signals, one directionally dependent association and one mutually independent association, or a pair of directionally dependent association signals. Figure S8: (a) Direction of effect for eSNP sources association with gene targets expression in (a) real data (b) permutations (c) permutations when the eSNP sou [file pgen.1004587.s002.docx]

**Co-regulated transcripts associated to cooperating eSNPs define bi-fan motifs in human gene networks** **- Kreimer *et al.***

**Supplementary Information**

**
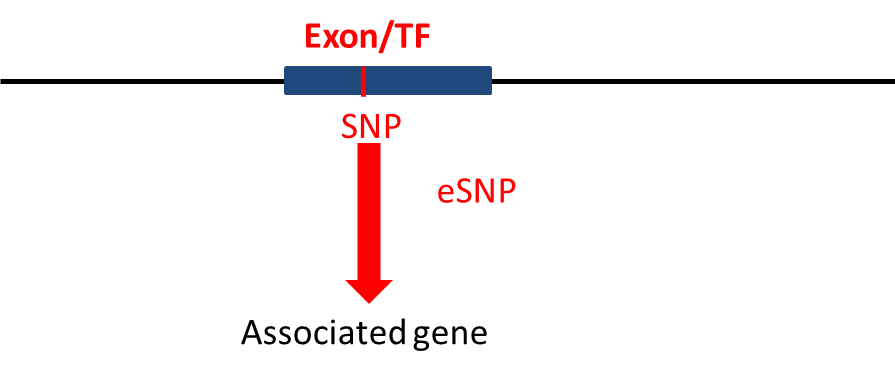
**

Figure S1: Illustrating the association testing between pairs of SNPs within known regulatory regions and genes (close or distal): regulatory elements appear in red. If the regulatory element has a SNP within the boundaries of an exon or a TF then we check for association (*p* < 10^-4^ denoted by a red edge) using linear regression between the minor allele count of the SNP and any gene.


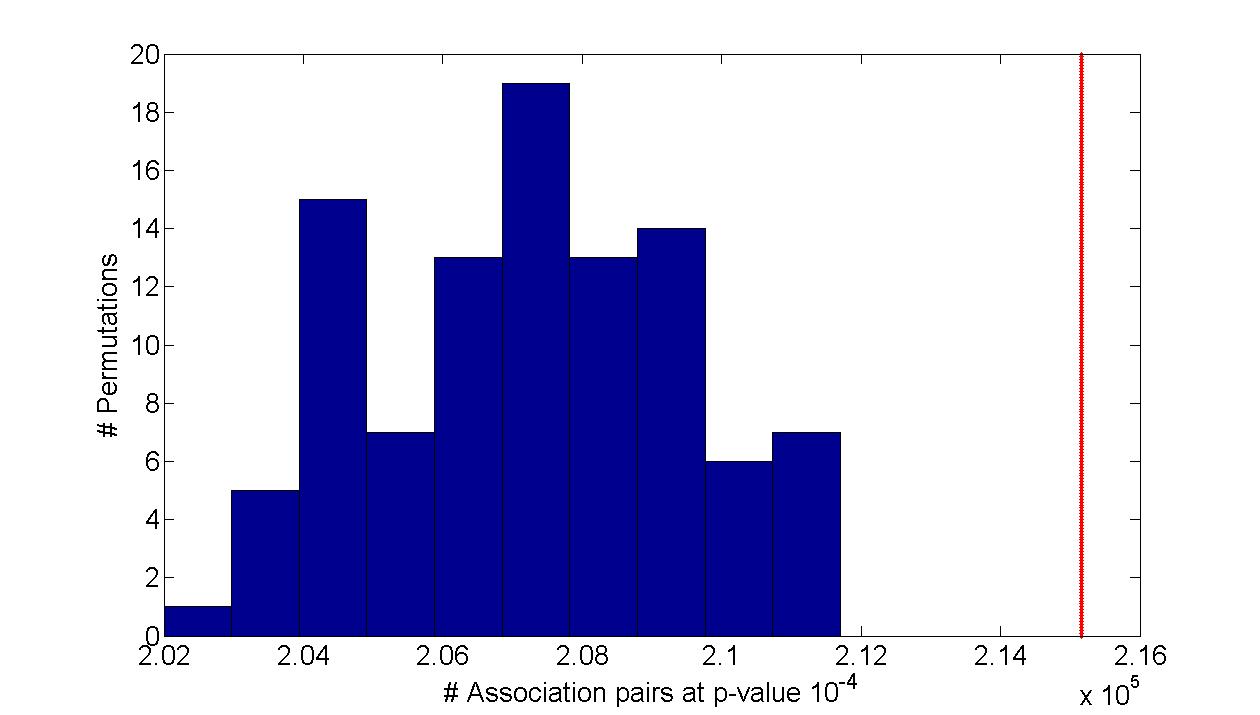


Figure S2: Histogram of the number of association pairs in 100 permutations for a p-value cutoff 10^-4^. The red line indicates this number in the real data.


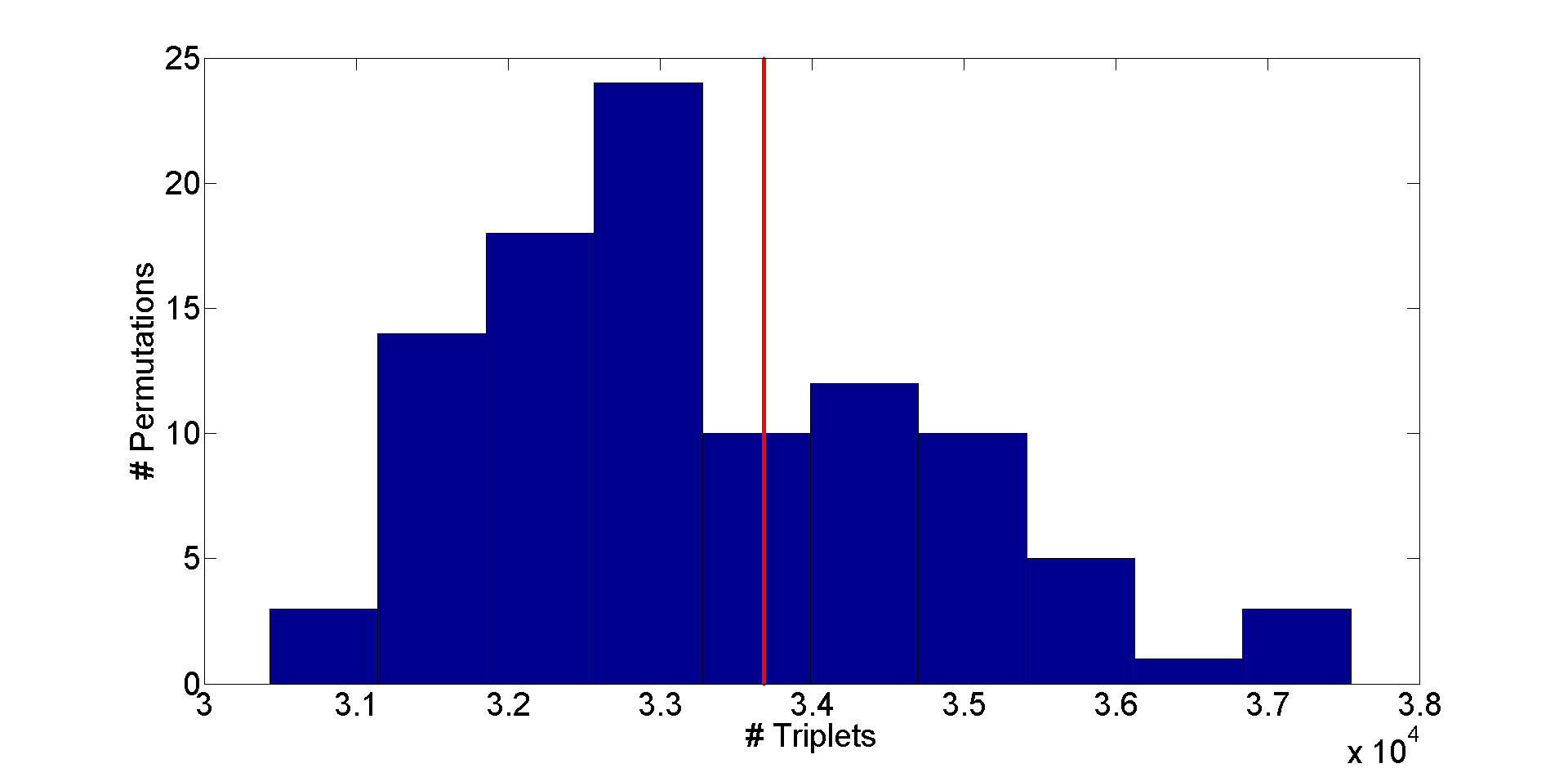


Figure S3: Histogram of the number of triplets in 100 permutations, at association p-value of 10^-4^. The red line indicates the observed number of triplets in real data at association p-value 10^-4.52^.


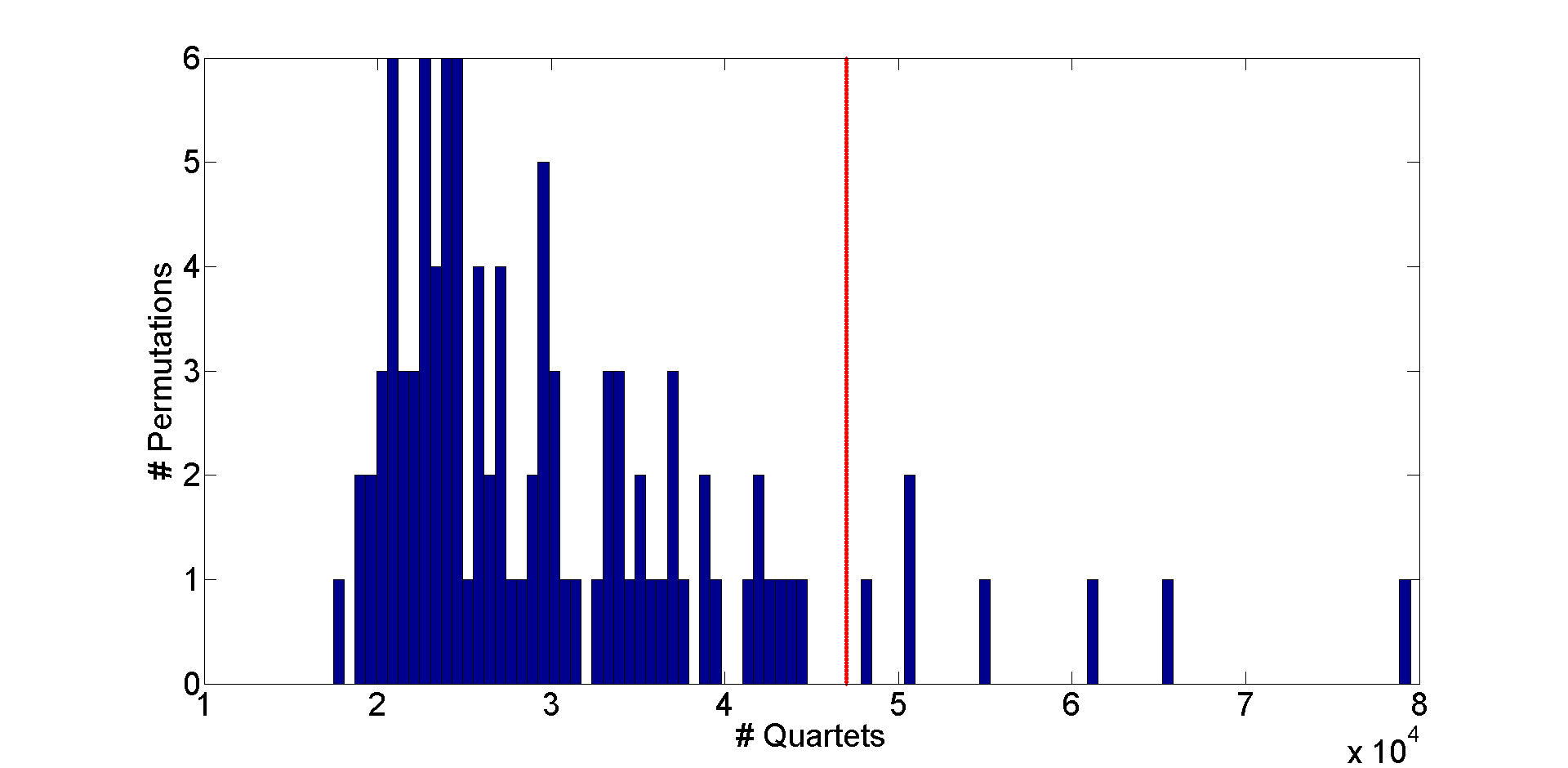


Figure S4: Histogram of the number of quartets in 100 permutations, at association p-value of 10^-4^. The red line indicates the observed number of quartets in real data at association p-value 10^-4.52^.


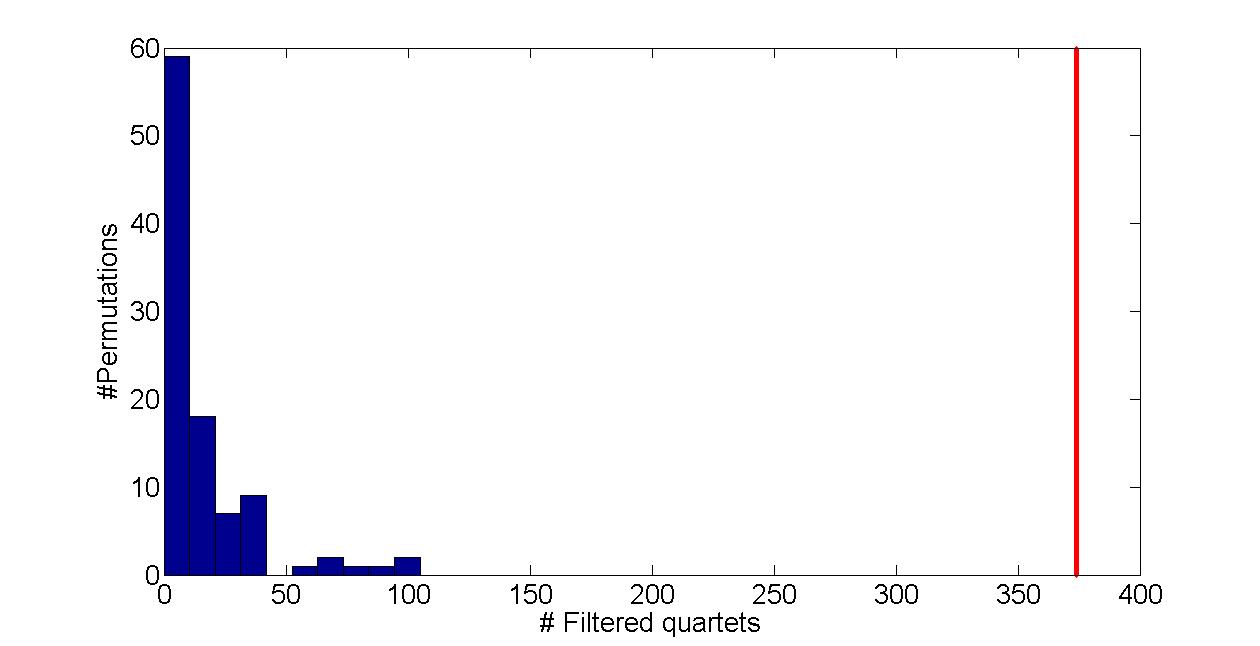


Figure S5: Histogram of the number of filtered quartets in 100 permutations, at association p-value of 10^-4^. The red line indicates the observed number of filtered quartets in real data at association p-value 10^-4.52^.


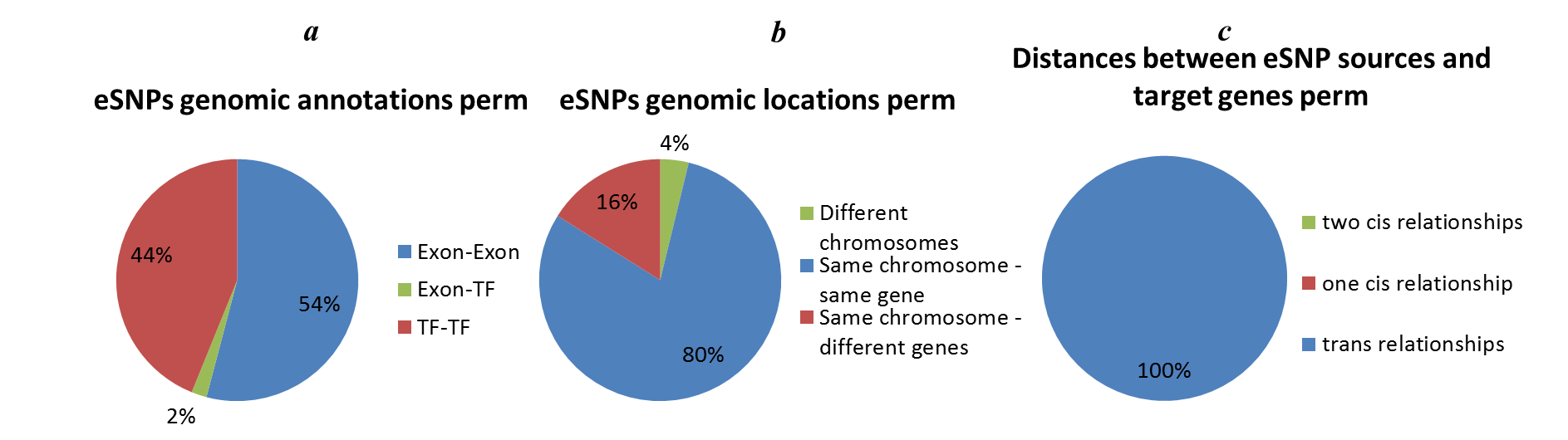


Figure S6: Distribution of genomic properties of eSNP sources in the permuted set: by (a) genomic annotation (b) relative genomic location (c) distances between them and their targets. An eSNP is said to be *in* *cis* if it resides within the span of the target gene and *in trans* otherwise.


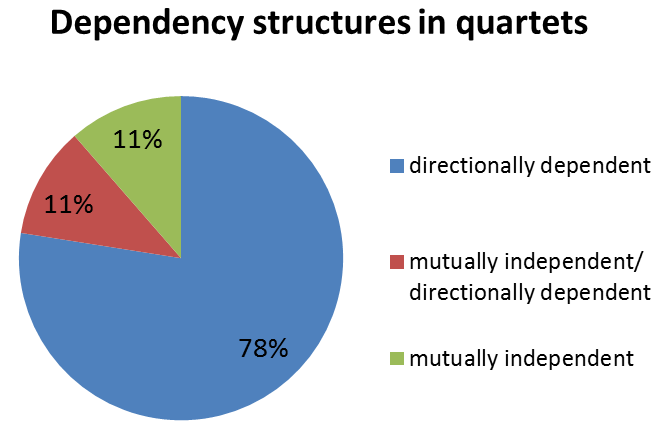


Figure S7: Dependency structures in quartets of the permuted set: Quartets are either comprised of a pair of mutually independent association signals, one directionally dependent association and one mutually independent association, or a pair of directionally dependent association signals.


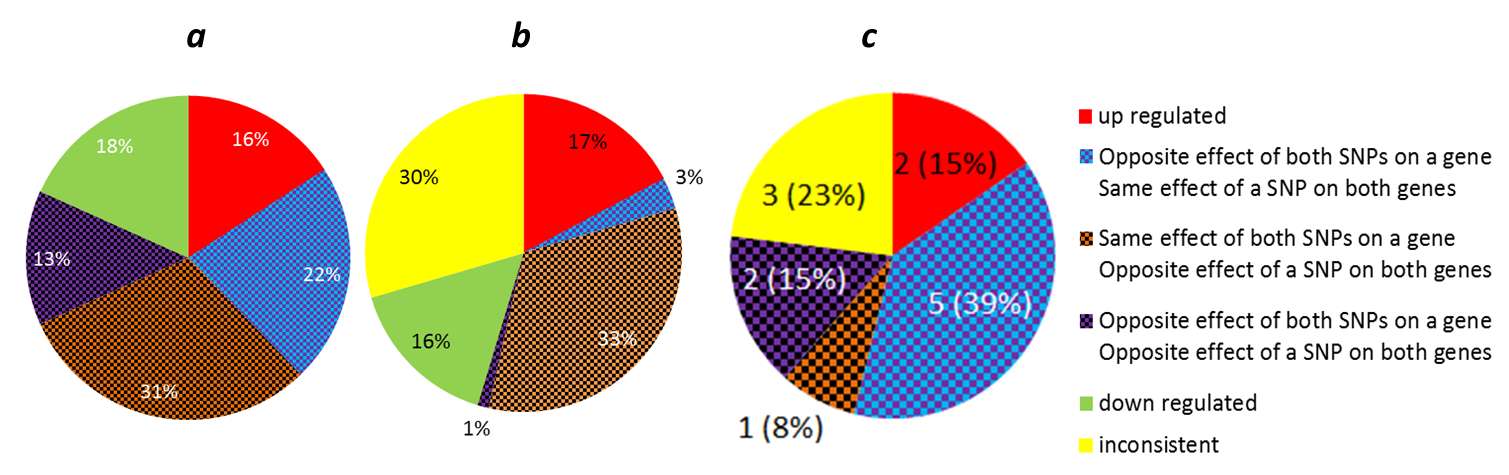


Figure S8: (a) Direction of effect for eSNP sources association with gene targets expression in (a) real data (b) permutations (c) permutations when the eSNP sources are located on different chromosomes. Both SNPs can have either the same or opposite effect on gene targets. The effect of a SNP on both genes is either the same or opposite.


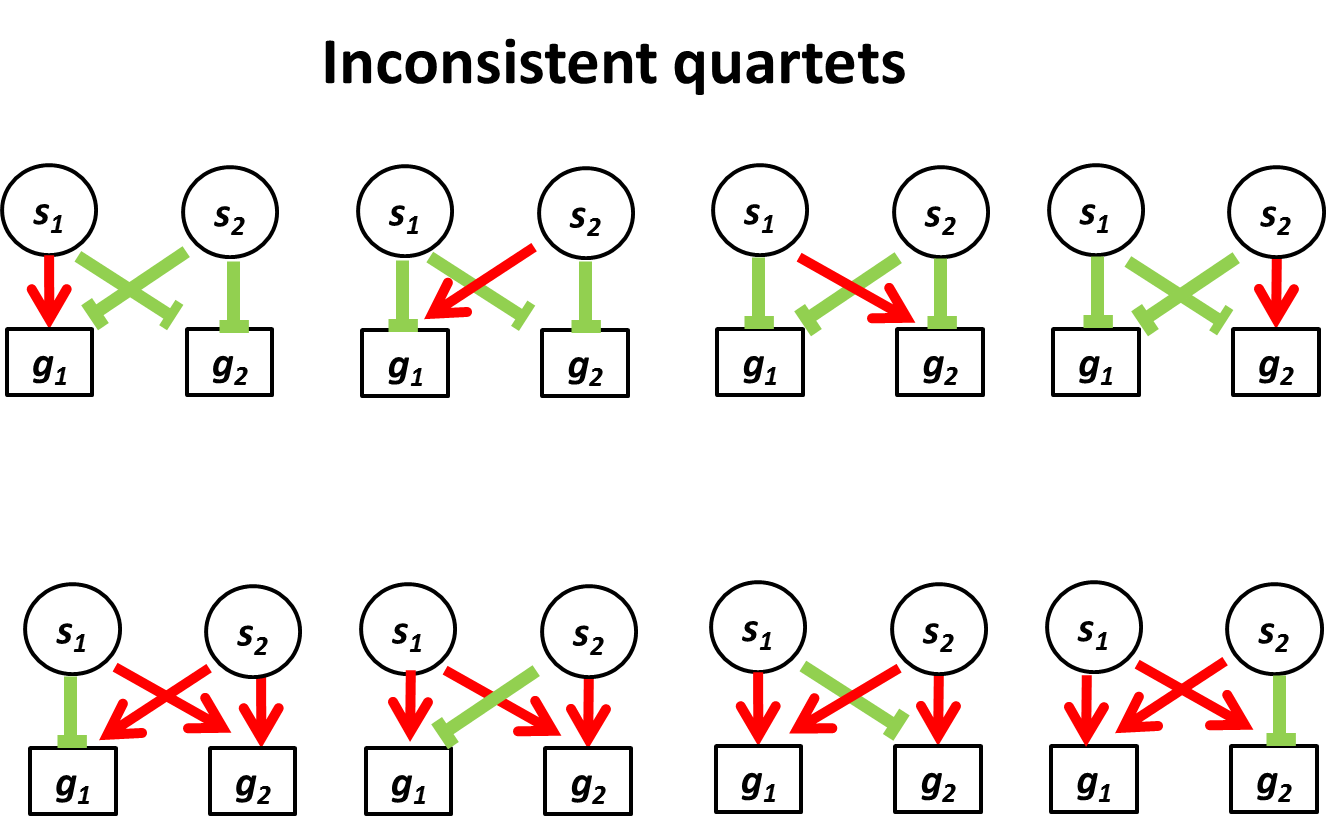


Figure S9: All eight regulation patterns of inconsistent quartets.

Note S1: Statistical challenges in comparing real vs. permuted quartets.

There are a couple of statistical challenges involved in comparison of real quartets to those observed in permutations. One bias is that of proximal eSNP sources in permutations. This leads for example to the artifact of enrichment of opposite direction eSNP sources in real data, comparing to the proximal, hence correlated effect eSNPs in permutations (Figures S8a and S8b). A second challenge is due to the rarity of eSNP sources on different chromosomes in permutations. This makes it statistically hard for comparing characteristics of sub-groups between real and permuted data (Figures S8c).

Table S1: A comprehensive description of 82 cooperating quartets. 3000000000 correspond to different chromosomes.

| Pop | #associations 10^-5^ | #expected associations at 10^-5^ | #filtered quartets | Same effect of both eSNPs (%64) | Distal regulation (92%) | S_1_ –S_2_ diff chr (75%) | G_1_-G_2_ diff chr (83%) | Consistency  (100%) |
| --- | --- | --- | --- | --- | --- | --- | --- | --- |
| EUR | 50048 | 10287 | 21674 | 82% | 78% | 88% | 89% | 99.3% |
| CEU | 54232 | 10155 | 43341 | 99% | 88% | 77% | 92% | 99.9% |
| FIN | 43111 | 10334 | 16663 | 90% | 82% | 88% | 84% | 99.7% |
| GBR | 43396 | 10267 | 18398 | 98% | 84% | 92% | 82% | 99.9% |
| TSI | 44562 | 10251 | 16171 | 93% | 86% | 93% | 90% | 100% |
| YRI | 94671 | 14698 | 51115 | 96% | 85% | 87% | 91% | 99.9% |

Table S2: Replication of quartets’ properties in the Geuvadis dataset [[1](#_ENREF_1)]. For each property in the first row we indicate the percentage in the original, smaller dataset.

| SNP ID | Assembled sequences |
| --- | --- |
| rs9274634 | COX:AL662789.11_80240_AC  QBL:AL662789.11_80240_AC  MANN:AL662789.11_80240_AC  MCF:AL662789.11_80240_AC |
| rs1129740 | DBB:AL662789.11_53222_GA  COX:AL662789.11_53222_GA  QBL:AL662789.11_53222_GA  MANN:AL662789.11_53222_GA  SSTO:AL662789.11_53222_GA  MCF:AL662789.11_53222_GA |
| rs1142334 | DBB:AL662789.11_53388_GC  COX:AL662789.11_53388_GC  QBL:AL662789.11_53388_GC  MANN:AL662789.11_53388_GC  SSTO:AL662789.11_53388_GC  MCF:AL662789.11_53388_GC |
| rs9274389 | COX:AL662789.11_76776_CT  QBL:AL662789.11_76776_CT  MANN:AL662789.11_76776_CT |
| rs2808143 | DBB:AL662789.11_49324_CG  COX:AL662789.11_49324_CG  QBL:AL662789.11_49324_CG  MANN:AL662789.11_49324_CG  SSTO:AL662789.11_49324_CG  MCF:AL662789.11_49324_CG |
| rs1130034 | DBB:AL662789.11_49391_TC  COX:AL662789.11_49391_TC  QBL:AL662789.11_49391_TC  MANN:AL662789.11_49391_TC  SSTO:AL662789.11_49391_TC  MCF:AL662789.11_49391_TC |
| rs8227 | DBB:AL662789.11_54874_AG  COX:AL662789.11_54874_AG  QBL:AL662789.11_54874_AG  MANN:AL662789.11_54874_AG  SSTO:AL662789.11_54874_AG  MCF:AL662789.11_54874_AG |
| rs1130116 | DBB:AL662789.11_54652_AC  COX:AL662789.11_54652_AC  QBL:AL662789.11_54652_AC  MANN:AL662789.11_54652_AC  SSTO:AL662789.11_54652_AC  MCF:AL662789.11_54652_AC |
| rs9272851 | DBB:AL662789.11_54821_TC  COX:AL662789.11_54821_TC  QBL:AL662789.11_54821_TC  MANN:AL662789.11_54821_TC  SSTO:AL662789.11_54821_TC  MCF:AL662789.11_54821_TC |

Table S3: The distribution of HLA common variants in specifically assembled sequences.

References

1. Lappalainen T, Sammeth M, Friedlander MR, t Hoen PA, Monlong J, et al. (2013) Transcriptome and genome sequencing uncovers functional variation in humans. Nature 501: 506-511.
